# Hydrogen-substituted β-tricalcium phosphate synthesized in organic media

**DOI:** 10.1107/S2052520616015675

**Published:** 2016-12-01

**Authors:** Christoph Stähli, Jürg Thüring, Laëtitia Galea, Solène Tadier, Marc Bohner, Nicola Döbelin

**Affiliations:** a RMS Foundation, Bettlach, Switzerland; b Department of Materials, ETH Zürich, Zürich, Switzerland; c MATEIS, INSA-Lyon, Villeurbanne CEDEX, France; d Institute of Geological Sciences, University of Bern, Bern, Switzerland

**Keywords:** β-tricalcium phosphate, whitlockite, calcium deficiency, X-ray diffraction, Rietveld refinement, bone substitute

## Abstract

β-Tricalcium phosphate (β-TCP) platelets synthesized in ethylene glycol offer interesting geometries for nano-structured composite bone substitutes but were never crystallographically analyzed. In this study, powder X-ray diffraction and Rietveld refinement revealed a discrepancy between the platelet structure and the known β-TCP crystal model. In contrast, a model featuring partial H for Ca substitution and the inversion of P1O_4_ tetrahedra, adopted from the whitlockite structure, allowed for a refinement with minimal misfits and was corroborated by HPO_4_^2−^ absorptions in Fourier-transform IR spectra. The Ca/P ratio converged to 1.443 ± 0.003 (*n* = 36), independently of synthesis conditions. As a quantitative verification, the platelets were thermally decomposed into hydrogen-free β-TCP and β-calcium pyrophosphate which resulted in a global Ca/P ratio in close agreement with the initial β-TCP Ca/P ratio (ΔCa/P = 0.003) and with the chemical composition measured by inductively coupled plasma (ΔCa/P = 0.003). These findings thus describe for the first time a hydrogen-substituted β-TCP structure, *i.e.* a Mg-free whitlockite, represented by the formula Ca_21 − *x*_(HPO_4_)_2*x*_(PO_4_)_14 − 2*x*_, where *x* = 0.80 ± 0.04, and may have implications for resorption properties of bone regenerative materials.

## Abstract 2

A hydrogen substitution mechanism, previously unknown in pure β-tricalcium phosphate, was discovered in crystals precipitated from ethylene glycol solutions. The structure was described by means of Rietveld refinement of powder X-ray diffraction data and corroborated by chemical analysis and IR spectroscopy.

## 1. Introduction   

Calcium phosphates (CaPs) have been widely used as synthetic bone graft substitutes and exhibit excellent biocompatibility, osteoconductivity and a chemical composition similar to bone mineral (LeGeros, 2002 ▸). Sintered hydroxyapatite [HA, Ca_5_(PO_4_)_3_OH], β-tricalcium phosphate [β-TCP, Ca_3_(PO_4_)_2_], or biphasic blends of the two constitute the most common commercially available CaP materials. In particular, β-TCP is of interest owing to its cell-mediated resorbability *in vivo* (Bohner, 2010 ▸).

While sintering of CaPs results in a limited range of geometries and specific surface areas, wet-chemical synthesis methods open the door to nano-sized entities and have been extensively studied for their potential in a broad range of biomedical applications including tissue engineering, drug/gene delivery and the design of structured composites (Loomba & Bhupinder, 2015 ▸; Makarov *et al.*, 2010 ▸). In contrast to phases such as HA and brushite (CaHPO_4_·2H_2_O), β-TCP cannot be precipitated from aqueous solutions. Only in the presence of Mg, whitlockite [*e.g.* Ca_18_Mg_2_(HPO_4_)_2_(PO_4_)_12_], a naturally occurring mineral with a crystal structure equivalent to that of β-TCP can be synthesized at ambient or hydrothermal conditions (Hamad & Heughebaert, 1986 ▸). Nevertheless, micron- or nano-sized (Mg-free) β-TCP particles have been obtained by precipitation in aqueous medium under autoclave conditions (Toyama *et al.*, 2002 ▸; Galea *et al.*, 2015 ▸) as well as in methanol (Bow *et al.*, 2004 ▸) and ethylene glycol (Tao *et al.*, 2008 ▸, 2009 ▸; Galea *et al.*, 2013 ▸, 2014 ▸).

Precipitation in ethylene glycol between 363 and 443 K allowed for the synthesis of sub-micrometric uniform hexagonal β-TCP platelets with controllable geometries (Galea *et al.*, 2013 ▸, 2014 ▸). Specifically, by modifying synthesis parameters including the solution concentration and acidity, the aspect ratio could be varied in the range of 1 to 14 with size dispersions as low as 5%. Since CaP ceramics are inherently brittle, platelets with high aspect ratios are of particular interest as fillers in nano-structured ceramic polymer composites inspired by natural materials such as nacre, which may provide suitable properties for load-bearing bone substitutes (Tang *et al.*, 2003 ▸). Moreover, owing to their non-agglomerating properties, crystals with an aspect ratio of 1 may enhance the flowability of CaP pastes or cements.

X-ray diffraction (XRD) patterns of β-TCP platelets previously revealed a poor agreement with the known β-TCP crystal model (Galea *et al.*, 2013 ▸; Dickens *et al.*, 1974 ▸). Recently, preliminary analysis of the platelet crystal structure indicated a sub-stoichiometric Ca-occupation. The crystal structure of a ceramic strongly influences properties including the solubility, which in turn determines the *in vivo* resorption of a bone graft. In particular, the dissolution rate and bone bonding properties of Ca-deficient HA [CDHA, Ca_10 − *x*_(HPO_4_)_*x*_(PO_4_)_6 − *x*_(OH)_2 − *x*_, where 0 < *x* ≤ 1] were shown to depend on its Ca/P ratio (Radin & Ducheyne, 1993 ▸; Mavropoulos *et al.*, 2003 ▸). Therefore, in order to successfully apply organic media-synthesized β-TCP platelets for bone regeneration, their crystal structure, and in particular the Ca-deficiency, must be well understood.

In this study, β-TCP platelets were examined by Rietveld refinement of powder XRD patterns as well as by Fourier-transform IR (FTIR) spectroscopy and chemical analysis. Furthermore, the effect of synthesis parameters on the platelet stoichiometry was investigated. Finally, the platelet crystal structure was discussed in comparison with sintered β-TCP and with synthetic Mg-whitlockite.

## 2. Materials and methods   

### 2.1. Syntheses   

β-TCP platelets were produced by adapting a previously reported precipitation method (Tao *et al.*, 2008 ▸), either in a batch reactor as recently described (Galea *et al.*, 2013 ▸), or in a continuous tubular reactor. Briefly, a CaCl_2_–ethylene glycol solution was mixed with a H_3_PO_4_ (or Na_2_HPO_4_)–ethylene glycol solution, pH-adjusted by NaOH and kept at constant temperature (363 to 443 K) for at least 30 min. A detailed description of the synthesis of β-TCP platelets is given in the supporting information §S1.1 and an example of the resulting morphology is shown in §S2.1.

Reference β-TCP materials were produced through sintering of CaCO_3_ (CaCO_3_, MERCK, Germany) and monetite (CaHPO_4_, GFS Chemicals, USA) at 1173 to 1273 K, resulting in 100% β-TCP (used for FTIR analysis) or 93 wt% β-TCP and 7 wt% hydroxyapatite (HA, used for elemental analysis). Mg-whitlockite was synthesized hydrothermally by incubating 1 g of monetite with 20 ml of a 1.5 m*M* MgCl_2_ solution at 473 K for 1 d in a steel autoclave lined with a Teflon capsule (inner volume of 45 ml).

### 2.2. Crystallographic analysis   

The crystal structure of the synthesized platelets was studied by means of powder X-ray diffraction (XRD). Samples were inserted into a glass capillary (diameter: 0.5 mm, glass type no 10; Hilgenberg GmbH, Germany) aligned and rotating on the goniometer axis in a Bruker D8 Advance diffractometer (Bruker AXS GmbH, Germany). XRD patterns were collected using digitally and Ni-filtered Cu *Kα* radiation (wavelength: 1.540598 Å) in transmission geometry from 5 to 60° 2θ at a step size of 0.012° and an acquisition time of 5.75 s per step.

The resulting patterns were analyzed by Rietveld refinement (Rietveld, 1969 ▸) using *BGMN* software, Version 4.2.22 (Bergmann *et al.*, 1998 ▸) and Profex user interface, Version 3.9.2 (Doebelin & Kleeberg, 2015 ▸). Since varying fractions of monetite were present as a by-product of the β-TCP synthesis, a monetite phase model (Dickens *et al.*, 1972 ▸), PDF# 04-009-3755, was included in the refinement. Moreover, a broad signal at around 32° (2θ) was detected in some samples and was attributed to a nanocrystalline chlorapatite phase, although no unambiguous identification of the type of apatite was possible. Consequently, the chlorapatite phase model (Hughes *et al.*, 1989 ▸), PDF# 04-012-1323, was included, which improved the quality of the fits. The β-TCP structure (Dickens *et al.*, 1974 ▸), PDF# 04-008-8714, was taken as a starting model and modified to better fit the observed patterns, as elaborated in §3. The refinement was verified to be independent of the extent of measurement noise by comparison of two diffractograms obtained on the same sample using an acquisition time of 5.75 and 19 s per step, respectively. For Mg-doped platelets, the Mg fraction determined by elemental analysis was used to account for the lower electron density on the Ca5 position occupied by Mg atoms (Enderle *et al.*, 2005 ▸). In the case of synthetic whitlockite, the Ca5 position was fully occupied with a refined Mg/Ca ratio. The Ca4 site occupancy was refined as partially occupied with Ca, as proposed previously (Calvo & Gopal, 1975 ▸).

In order to monitor temperature-induced phase separations, *in-situ* XRD patterns were acquired using a heating chamber (Anton Paar HTK 1200, Anton Paar GmbH, Austria) in an X’Pert diffractometer in reflective geometry (X’Pert Pro MPD, Panalytical, The Netherlands) using Ni-filtered Cu *K*α radiation. Specifically, the temperature was raised in steps of 323 K from 773 to 1273 K and kept for 1 h at each step. For the quantitative phase analysis after calcination, samples were heated directly to 1273 K (heating rate: 17 K min^−1^) and kept at 1273 K for 1 h (or 1328 K for 48 h in the case of whitlockite). The resulting XRD patterns (obtained in transmission geometry as described earlier) were refined including the β-calcium pyrophosphate (β-CPP, Ca_2_P_2_O_7_) phase model (Boudin *et al.*, 1993 ▸), PDF# 04-009-3876. The HA phase model (Sudarsanan & Young, 1969 ▸), PDF# 01-074-0565, was included for the sintered reference material.

### 2.3. FTIR spectroscopy   

Optically clear pellets of 13 mm diameter were prepared by grinding and mixing 300 mg KBr (KBr, Uvasol^®^, MERCK, Germany) with approximately 1 mg of sample and subsequent pressing at 10 T for 2.5 min under vacuum. Transmission FTIR spectra were obtained on a Bruker Lumos IR spectrometer between 400 and 4000 cm^−1^ at a resolution of 4 cm^−1^ and 64 accumulations.

### 2.4. Elemental analysis   

In order to quantify the elemental composition, samples were dissolved in HNO_3_ (69% w/w, Trace SELECT, Fluka, Switzerland) and diluted 1:100 in demineralized H_2_O with a final concentration of 3% HNO_3_. Ca, P, Na and Mg concentrations were measured (*n* = 6 per sample) using inductively coupled plasma–mass spectroscopy (ICP-MS; Agilent 7700x, Agilent Technologies, Japan). ^44^Ca, ^31^P, ^23^Na and ^24^Mg signals were calibrated against certified single element standard solutions (Inorganic Ventures, USA) serially diluted to the following concentrations: (i) 100, 10 and 1 p.p.b. Na and (ii) 5000, 1000, 200 and 40 p.p.b. Ca combined with proportional P and Mg concentrations in a Ca:P:Mg weight ratio of 10:5:1. Additionally, calibration drifts were corrected according to a Ca–P–Mg standard measured after every 8th sample and according to a 40 p.p.b internal In/Sc/Bi standard solution (Inorganic Ventures, USA) measured along with each sample.

Since the preparation of ICP standard solutions involves pipetting errors (approximately ± 2%), the Ca/P ratio was corrected ((Ca/P)_ICP,corr_) based on a chemically pure, high-temperature sintered β-TCP/HA reference material. Specifically, the difference between the Ca/P ratios measured in the reference through XRD, by phase fraction quantification assuming stoichiometric phases (Ishikawa *et al.*, 1993 ▸), and through ICP (Ca/P = 1.512 and 1.486, respectively) was subtracted from all Ca/P ratios. (The sum of the Na/P, Mg/P and Sr/P molar ratios in the reference was verified to be below 0.002.)

## 3. Results   

### 3.1. Crystal structure model for Rietveld refinement   

**Figure 1:**
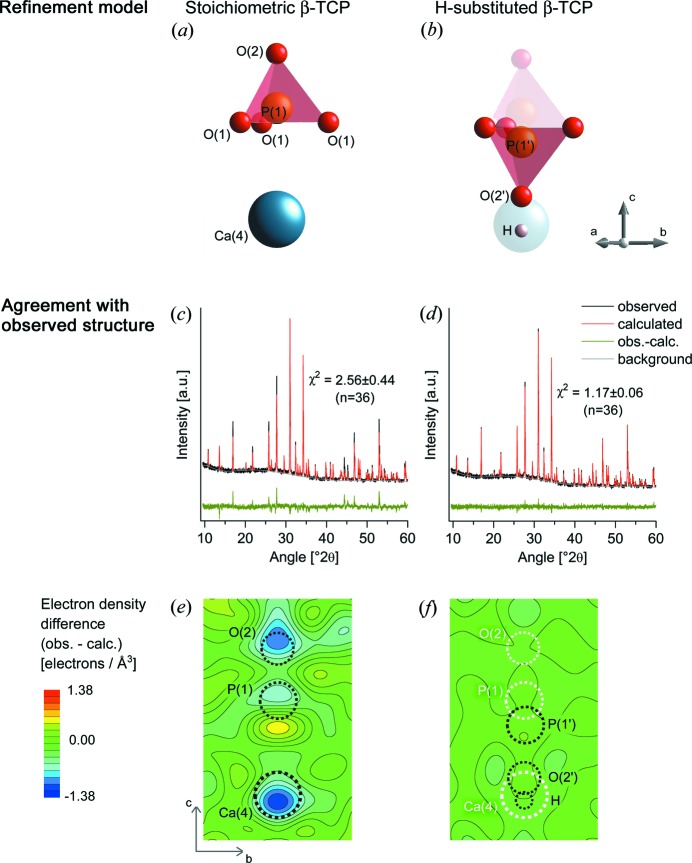
Illustration of the Ca4 and P1O_4_ atomic arrangement in (*a*) the stoichiometric β-TCP crystal model (Dickens *et al.*, 1974 ▸) and (*b*) a hydrogen-substituted β-TCP model where some of the P1O_4_ tetrahedra are inverted and protonated. Representative XRD patterns of β-TCP platelets fitted by Rietveld refinement with (*c*) the stoichiometric and (*d*) the hydrogen-substituted crystal model. (Monetite and chlorapatite fractions were negligible in this sample and were not refined for the purpose of this illustration.) The difference (green line) between the observed (black) and calculated (red) intensity, characterized by χ^2^ values, was larger for the stoichiometric compared with the hydrogen-substituted model. EDD maps between the observed structure and (*e*) the structure calculated with the stoichiometric model indicated sub-occupied O2, P1 and Ca4 sites along with a positive region immediately below the P1 site, while (*f*) using the hydrogen-substituted model resulted in much smaller EDDs.

The Ca4 and P1O_4_ region of the stoichiometric β-TCP crystal structure (Dickens *et al.*, 1974 ▸) is shown in Fig. 1 ▸(*a*). This structure was used as a starting model to fit XRD patterns of β-TCP platelets produced in ethylene glycol. Applying this model confirmed space group *R*3*c* but, although refining unit-cell dimensions, scale factor, crystallite size, microstrain and texture, resulted in substantial misfits of relative peak intensities between observed and calculated patterns (Fig. 1 ▸ *c*). Difference-Fourier maps were generated to visualize the misfits in direct space electron densities using the software *Promap* (Doebelin; unpublished addon for *Profex*; Doebelin & Kleeberg, 2015 ▸). Most atoms showed slight displacements, which could be refined with stable convergence by defining the P2O_4_ and P3O_4_ tetrahedra as rigid bodies with the dimensions reported in the stoichiometric β-TCP model (Dickens *et al.*, 1974 ▸) and with refined translation and rotation. In contrast, major discrepancies were observed for several atoms with coordinates 0,0,*z*. Namely, the site occupancies of the Ca4 and O2 positions were lower than the stoichiometric values (0.5 and 1.0, respectively), as revealed by negative differences between observed and calculated electron densities (Fig. 1 ▸ *e*). Moreover, a negative difference at the P1 site and an adjacent positive region (slightly closer to the Ca4 site) indicated the splitting of this position into two sites. Based on the elemental analysis (see §3.4), the lower Ca4 site occupancy cannot be explained by substitution with lighter Mg atoms. However, the observed arrangement showed similarities to the crystal structure of whitlockite, previously described using single-crystal XRD structure refinements (Calvo & Gopal, 1975 ▸). In their model, the Ca4 position was sub-occupied and the P1O_4_ tetrahedron (comprising P1, three O1 and one O2 atom) was protonated and mirrored about the O1 base plane, resulting in a new P1′ position and a new O2′ site connected to a H atom (Fig. 1 ▸ *b*).

The resolution of the powder XRD data was not sufficient to unambiguously identify individual atomic species and site occupancies simultaneously. In particular, since the O2′H group of the flipped tetrahedron strongly overlapped with residual Ca on the Ca4 position, independent refinement of Ca4 deficiency and the number of flipped tetrahedra was not possible. However, adopting the model of inverted tetrahedra (Calvo & Gopal, 1975 ▸) allowed restrictions to be applied to eventually describe the structure by refining one single parameter. Specifically, as the tetrahedra could only flip as a whole, the fraction of inverted P1O_4_ tetrahedra, *f_m_*, was defined and linked to the individual site occupancies, *P*, as follows: *P*_P1′_ = *P*_O2′_ = *P*_H_ = *f_m_* and *P*_P1_ = *P*_O2_ = 1 − *f_m_*. Moreover, in order to maintain charge balance, two tetrahedra were inverted and protonated for each missing Ca^2+^ ion, *i.e. P*_Ca4_ = (1 − *f_m_*)/2. The resulting model for refined site occupancies also restricted the overlapping sites *P*_Ca4_ + *P*_O2′_ to a maximum total occupancy of 1. Thus, taking into account all atomic positions in the β-TCP unit cell (6 P1 positions for 42 P positions in total), the general formula of the structure is β-Ca_21 − *f*_ *_m_*(HPO_4_)_2*f*_ *_m_*(PO_4_)_14–2*f*_ *_m_*. The P1O_4_ tetrahedra were treated as semi-rigid bodies by applying the following restrictions: the *z*-distance *z*_P1′_ − z_O1_ was linked to *z*_P1_ − *z*_O1_, while *z*_P1′_ − *z*_O2′_ and *z*_P1_ − *z*_O2_ were set equal to 0.1498 nm (Dickens *et al.*, 1974 ▸) and z_O2′_ − *z*_H_ to 0.0942 nm (Calvo & Gopal, 1975 ▸). The *x*_O1_ and *y*_O1_ coordinates could be refined without resulting in extensive distortion of the tetrahedra. Temperature factors were taken from the stoichiometric β-TCP model (Dickens *et al.*, 1974 ▸) and multiplied by a refined scale factor common for each type of atom.

This model was first validated by refining an XRD pattern of hydrothermally synthesized Mg-whitlockite, which resulted in a good fit (χ^2^ = 1.11; defined previously; Toby, 2006 ▸) and a stable convergence to an *f_m_* value of 0.932 ± 0.009, *i.e. P*_Ca4_ = 0.034 ± 0.004 and (Ca + Mg)/P = 1.433 ± 0.001, where the errors represent the estimated standard deviation (e.s.d.) calculated by the refinement algorithm. This stoichiometry thus corresponds well with the theoretical composition of synthetic Mg-whitlockite: Ca_18_Mg_2_(HPO_4_)_2_(PO_4_)_12_, (Ca + Mg)/P = 1.429 (Gopal *et al.*, 1974 ▸). Note that the composition was also closely matched by ICP-MS analysis [(Ca/P)_ICP,corr_ = 1.265, (Mg/P)_ICP_ = 0.156, *i.e.* ((Ca + Mg)/P)_ICP_ = 1.421]. Moreover, the refinement confirmed that the Ca5 position in synthetic whitlockite was occupied exclusively by Mg which is in line with the theoretical composition and previous studies (Enderle *et al.*, 2005 ▸).

Using the same model to refine the structure of β-TCP platelets grown in organic media allowed for much better fits compared with the published model, as reflected in lower χ^2^ values (detailed in Table 1 ▸) and smaller electron density differences (EDDs; Figs. 1 ▸ *d* and *f*). The *f_m_* value thereby converged to 0.801 ±0 .041 (average and SD over 36 samples), corresponding to a *P*_Ca4_ of 0.100 ± 0.021 and a β-TCP Ca/P ratio of 1.443 ± 0.003. (The dependence of the stoichiometry on synthesis parameters is §3.5.) Table 2 ▸ summarizes the site occupancies as well as the atomic coordinates of the Ca4 position and the original and inverted (H)P1O_4_ tetrahedra. All refined structures and diffraction raw data are provided in crystallographic information files (CIF) as supporting information along with a sample list (Table S2) matching the synthesis condition numbers with the name of the datablocks in the CIFs.

### 3.2. FTIR analysis   

**Figure 2:**
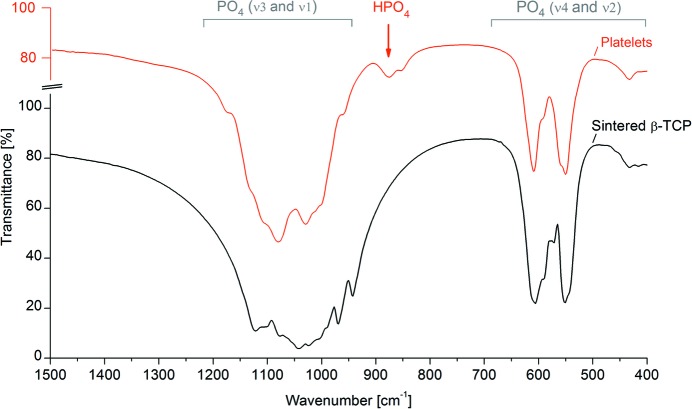
Transmission FTIR spectra of sintered β-TCP and β-TCP platelets. The phosphate absorption regions show several differences in relative peak intensity and/or peak shifts between the two materials. Platelets but not sintered β-TCP exhibited an absorption band at 875 cm^−1^, attributable to HPO_4_^2−^ groups.

The phosphate absorption region in the FTIR spectra of sintered β-TCP as well as β-TCP platelets is shown in Fig. 2 ▸. (The full wavenumber range is presented in the supporting information §S2.2; Fig. S2). The absorption bands observed in sintered β-TCP are consistent with previous reports (Jillavenkatesa & Condrate, 1998 ▸; Berzina-Cimdina & Borodajenko, 2012 ▸; Bigi *et al.*, 1997 ▸). Specifically, the bands at approximately 1120, 1105, 1080, 1042 and 1025 cm^−1^ can be assigned to the ν_3_ vibrational mode of the PO_4_^3−^ ion. Moreover, ν_1_-PO_4_ bands were observed at 970 and 942 cm^−1^, ν_4_-PO_4_ bands at 605, 592, 545 and 552 cm^−1^ and two weak ν_2_-PO_4_ bands at 415 and 435 cm^−1^. Additional weak absorptions, *e.g.* at 572 cm^−1^, also agree with previously reported β-TCP spectra (Bigi *et al.*, 1997 ▸).

The PO_4_ absorption bands in spectra of β-TCP platelets differ significantly from sintered β-TCP. Specifically, several bands in the ν_3_, ν_4_ and ν_2_ region may have undergone slight chemical shifts compared with sintered β-TCP or either appeared or disappeared due to changes in relative intensities. The two ν_1_ absorption bands disappeared in β-TCP platelets while new shoulders were observed at 960 and 1175 cm^−1^ (not matched by previously reported data). The absorption band at 875 cm^−1^ can be assigned to HPO_4_^2−^ groups, as previously observed in CDHA before sintering (Lin *et al.*, 1998 ▸, 2001 ▸; Cantwell *et al.*, 2014 ▸; Durucan & Brown, 2000 ▸). The shoulder at 855 cm^−1^ coincides with previous attributions to HPO_4_^2−^ groups in β-TCP synthesized under autoclave conditions (Toyama *et al.*, 2002 ▸) and in synthetic whitlockite (LeGeros *et al.*, 1989 ▸). Although CO_3_ absorptions have been reported close to 875 cm^−1^, the presence of CO_3_ species in the platelets can be ruled out due to the absence of any absorption bands in the 1420–1450 cm^−1^ region (Fowler, 1974 ▸; Sader *et al.*, 2013 ▸). Moreover, comparison of the spectra of β-TCP platelets to those of pure monetite (CaHPO_4_) and to platelet samples containing high monetite fractions demonstrated that the HPO_4_^2−^ signal did not originate from the monetite phase (elaborated in the supporting information §S2.2; Fig. S3). Similarly, the intensity of the HPO_4_^2−^ signal was verified to be independent of the fraction of chlorapatite in the sample. Finally, comparison of spectra before and after calcination at 673 K, as well as of pure ethylene glycol and ethanol confirmed that no signals from organic residues were detectable in the platelets. Therefore, these findings corroborate the H for Ca substitution in the β-TCP phase indicated by XRD analysis.

### 3.3. Quantification of thermally induced phase changes   

**Figure 3:**
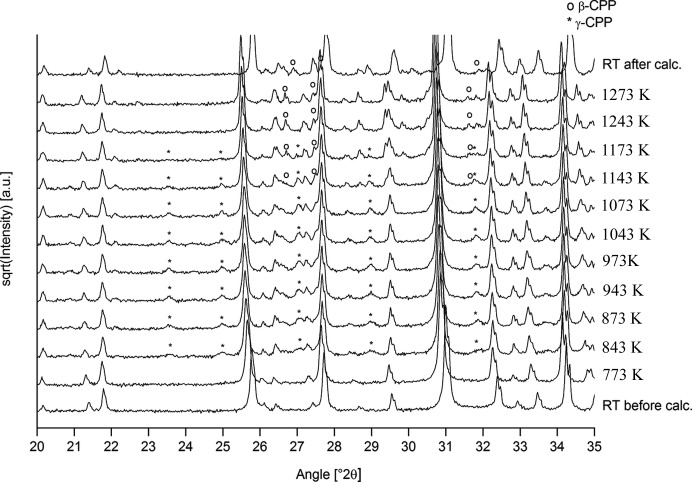
XRD patterns of β-TCP platelets before, during and after calcination up to 1273 K (RT: room temperature). Note that peak shifts are due to thermal expansion of the crystal lattice. In addition to the predominant β-TCP phase (non-labelled peaks), γ-CPP was observed between 823 and 1123 K whereas β-CPP appeared at 1123 K and also remained stable up to 1273 K after cooling to room temperature.

In order to examine the thermal stability of the Ca-deficient β-TCP phase, XRD patterns were acquired during and after calcination, which revealed the presence of γ-CPP (PDF# 00-017-0499) above 773 K and β-CPP (PDF# 04-009-3876) between 1073 and 1273 K as well as after returning to room temperature (Fig. 3 ▸). Note that the patterns obtained *in situ* during stepwise heating could not be refined due to the unknown crystal structure of the γ-CPP phase. Extensive peak shifts due to thermal expansion of the unit cells and the sample holder, the latter resulting in a sample height displacement error, were also observed. The Ca/P ratios as well as phase fractions determined by Rietveld refinement before and after calcination at 1273 K are given in Table 3 ▸. Before calcination, the overall Ca/P ratio (1.437 ± 0.003), calculated based on the weight fraction and molecular mass of each phase, was slightly lower than the refined β-TCP Ca/P ratio (1.445 ± 0.001) due to the presence of monetite (Ca/P = 1.0). After calcination, the refinement determined a β-TCP Ca/P ratio equal to the stoichiometric value of 1.5. This increase in the β-TCP Ca/P ratio was compensated for by the appearance of approximately 10 wt% β-CPP (Ca/P = 1.0, Table 3 ▸), where the resulting overall Ca/P ratio was in close agreement with the overall Ca/P ratio determined before calcination (difference: 0.2%). In summary, the thermal treatment induced a phase separation of Ca-deficient hydrogen-substituted β-TCP, along with the small quantities of monetite, into stoichiometric β-TCP and β-CPP, while maintaining the bulk Ca/P ratio. Good agreement of the Ca/P ratios determined from stoichiometric phase quantities after thermal treatment and from the structure refinement of hydrogen-substituted β-TCP prior to calcination thus corroborates the accuracy of the hydrogen-substituted refinement model.

### 3.4. Elemental composition   

The elemental composition of the platelets was assessed by ICP-MS in order to provide a second verification of the Rietveld refinement results (Table 4 ▸). Along with Ca and P concentrations (corrected as described in §2.4), Na and Mg concentrations were quantified because of the addition of NaOH to the reaction and due to possible Mg traces in the CaCl_2_ precursor. Calcination at 1273 K resulted in an increase of the Ca/P ratio and a strong decrease of the Na/P ratio, which is possibly related to the elimination of Na^+^ and PO_4_^3−^ ions bound to volatile organic residues on the crystal surface, although below the sensitivity of FTIR analysis as stated earlier. In particular, covalent bonding between phosphates and ethylene glycol chains has been reported previously (Penczek *et al.*, 2015 ▸).

The small quantities of Na and Mg atoms measured after calcination were likely present in the crystal structure of the as-synthesized platelets where they are known to substitute for Ca (Enderle *et al.*, 2005 ▸; Yoshida *et al.*, 2006 ▸). These atoms thus contribute to the total electron density which is interpreted as Ca occupancy by the refinement model described in §3.1 (except for Mg-doped platelets and whitlockite). Note that, based on the atomic number, charge and the concentration of Na and Mg cations determined by ICP, their influence on the determination of the *f_m_* value by the refinement was verified to be negligible. For comparison with the Ca/P ratio determined by XRD, a Ca-equivalent ratio, (Ca_eq_/P)_ICP,corr_, taking into account these trace elements, was calculated according to the number of electrons per cation, *i.e.* (Ca_eq_/P)_ICP,corr_ = (Ca/P)_ICP,corr_ + 10/18 × (Na/P)_ICP_ + 10/18 × (Mg/P)_ICP_. (The suffix ‘corr’ is explained in §2.4.) Before calcination, (Ca_eq_/P)_ICP,corr_ was significantly lower than (Ca/P)_XRD_, which is plausible if some PO_4_^3−^ ions were present outside the crystalline phase. In contrast, after calcination there was only a small difference between ICP and XRD values (0.2%), which was lower than the standard deviation over the three samples.

### 3.5. Effect of synthesis conditions   

**Figure 4:**
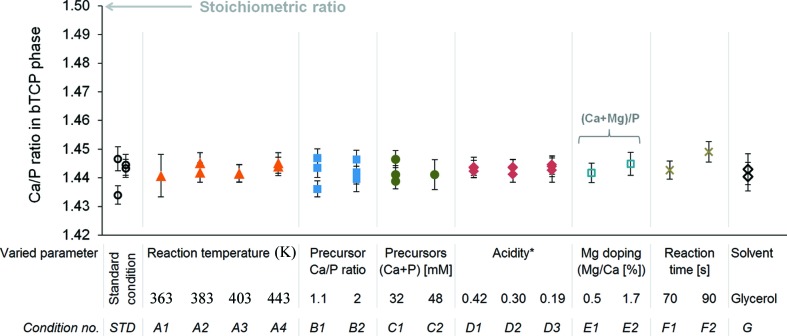
β-TCP Ca/P ratios determined by Rietveld refinement with the hydrogen-substituted model. The platelet synthesis conditions are detailed in Table S1. Error bars designate two times the e.s.d. (95% confidence interval) determined by the refinement algorithm. The average Ca/P ratio was equal to 1.443 ± 0.003 (SD; *n* = 36) with no significant effect of any of the investigated synthesis parameters.

In order to investigate the role of synthesis conditions in the crystallization of the hydrogen-substituted structure, the reaction temperature, precursor Ca/P ratio and total concentration, acidity, Mg doping, reaction time and solvent type were varied (detailed in Table S1). None of the investigated synthesis parameters had a significant effect on the β-TCP Ca/P, or (Ca + Mg)/P, ratio determined by XRD and Rietveld refinement (Fig. 4 ▸). A statistical analysis of this data is elaborated in the supporting information §S2.3.

## 4. Discussion   

This study examined the particular features distinguishing the crystal structure of β-TCP platelets synthesized in ethylene glycol from high-temperature sintered, stoichiometric β-TCP. For this purpose, the structure was analyzed by means of Rietveld refinement of XRD patterns, along with IR spectroscopy and chemical analysis.

Refinement of the platelet structure using the published β-TCP crystal model (Dickens *et al.*, 1974 ▸) revealed significant discrepancies at the Ca4, P1 and O2 crystallographic positions. On the other hand, much better fits were achieved by adopting a model containing a Ca4 deficiency along with the inversion and protonation of P1O_4_ tetrahedra. Given the major changes in site occupancy factors at the Ca4 and O2 positions compared with stoichiometric β-TCP, some displacement of other atoms was expected. Therefore, the fractional coordinates of all atomic sites were refined while treating phosphate tetrahedra as rigid bodies with some translational and rotational freedom. These lattice distortions were in line with the observed differences in the PO_4_ absorption bands in FTIR spectra. Moreover, the refined unit-cell constants were slightly different (< 0.4%) from the published β-TCP model (Dickens *et al.*, 1974 ▸). These dimensions are in agreement with previous measurements of interplanar distances in ethylene glycol-synthesized β-TCP, which were not precise enough to detect the deviation from the published structure (Tao *et al.*, 2008 ▸).

While the resolution of powder diffraction data alone is not sufficient to distinguish between the presence of H atoms and vacancies, several findings corroborated the proposed model of inverted HPO_4_^2−^ groups. A preliminary model involving vacancies on both the Ca4 and O2 positions without further rearrangement was considered. However, the resulting fit with the observed structure was less precise compared with the model involving inverted HPO_4_^2−^ tetrahedra. The presence of HPO_4_^2−^ groups was further supported by the absorption band at 875 cm^−1^ observed in the FTIR spectra. Moreover, this model was previously shown to allow for successful refinement of a whitlockite crystal structure (Calvo & Gopal, 1975 ▸). Whitlockite crystallizes in space group *R*3*c* with a unit cell equivalent to that of β-TCP and can be described by the idealized formula Ca_18_(*M* ^2+^)_2_(HPO_4_)_2_(PO_4_)_12_, where *M* ^2+^ is a divalent cation substituting for Ca^2+^, typically Mg^2+^ (Calvo & Gopal, 1975 ▸; Jang *et al.*, 2014 ▸). Here, a hydrothermally synthesized Mg-whitlockite was refined with minimal mismatch using the described model, resulting in a stoichiometry closely matching the synthetic whitlockite formula. Therefore, the mechanism of the H for Ca substitution and inversion of the tetrahedron appear to be identical in both whitlockite and β-TCP platelets. The platelet crystal structure can thus be defined both as Mg-free whitlockite or, in other words, hydrogen-substituted β-TCP. However, in contrast to synthetic whitlockite, not all of the P1O_4_ tetrahedra were inverted in platelets (*f_m_* = 0.80 ± 0.04). Interestingly, a similar fraction has been reported in a naturally occurring whitlockite, described by the formula Ca_18.19_(Mg_1.17_Fe_0.83_)H_1.62_(PO_4_)_14_ (*i.e. f_m_* = 0.81), which can be explained by a solid solution of synthetic whitlockite and merrilite (*e.g.* Mg- and Na-substituted β-TCP) structure (Calvo & Gopal, 1975 ▸; Hughes *et al.*, 2008 ▸).

Whitlockite precipitates from aqueous solutions at ambient or hydrothermal conditions, provided that Mg is available (Hamad & Heughebaert, 1986 ▸). In contrast, in the absence of Mg, phases including brushite, CDHA or HA are more stable than the β-TCP structure (Dorozhkin & Epple, 2002 ▸). Therefore, Mg-free β-TCP was never observed to precipitate from aqueous solutions, but forms above 1073 K by decomposition of CDHA or by solid-state reactions (Gibson *et al.*, 2000 ▸). Nevertheless, pure β-TCP has been produced from amorphous calcium phosphate precursors under autoclave conditions at 493 K (Toyama *et al.*, 2002 ▸), while α-TCP was transformed at 423 to 473 K into a biphasic mixture of CDHA and up to 20% β-TCP (Galea *et al.*, 2015 ▸). A higher stability of β-TCP at elevated temperatures is in line with the fact that the solubility of β-TCP decreases more strongly with increasing temperature (between 298 and 363 K) compared with HA, brushite and monetite (Vereecke & Lemaître, 1990 ▸). Moreover, a higher temperature in ethylene glycol favored the precipitation of β-TCP over monetite (Galea *et al.*, 2013 ▸). Nevertheless, β-TCP nanoparticles have been synthesized at room temperature in methanol (Bow *et al.*, 2004 ▸), which underlines the importance of the solvent. In organic solvents, the precipitation of phases such as brushite or HA may be prevented because they require the presence of either H_2_O molecules or OH^−^ ions. The β-TCP materials synthesized through wet-chemical methods mentioned here were either not analyzed for H- for Ca-substitution (Galea *et al.*, 2015 ▸; Bow *et al.*, 2004 ▸) or exhibited only a small fraction of HPO_4_^2−^ groups, as revealed by FTIR, according to the formula Ca_2.98_(HPO_4_)_0.04_(PO_4_)_1.96_ (Toyama *et al.*, 2002 ▸). In contrast, the use of ethylene glycol and temperatures between 363 and 443 K resulted in much more significant hydrogen substitution.

Most CaP phases which precipitate in aqueous solution, *e.g.* CDHA, brushite or monocalcium phosphate monohydrate [MCPM, Ca(H_2_PO_4_)_2_·H_2_O], contain HPO_4_^2−^ (or H_2_PO_4_^−^) groups (Dorozhkin & Epple, 2002 ▸), which thus parallels the incorporation of HPO_4_^2−^ groups into platelets synthesized in ethylene glycol. At temperatures of 1273 K or more, CaP phases comprising HPO_4_^2−^ groups are known to be thermally unstable. In the case of CDHA with a variable degree of Ca deficiency, *x*, calcination leads to the following transformation
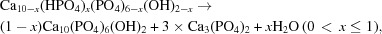
where quantification of the weight fractions of the biphasic calcined sample allows for accurate determination of the initial degree of Ca deficiency (Ishikawa *et al.*, 1993 ▸). The Ca-deficient β-TCP platelets decomposed into β-CPP (Ca/P = 1) and β-TCP that exhibited no detectable Ca-deficiency when refined with the same structure model and no longer showed a band at 875 cm^−1^ in FTIR spectra (data not shown). Hence, the thermal decomposition during calcination can be described as 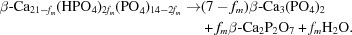
The as-determined overall Ca/P ratio of the biphasic sample coincided well with the refined Ca/P ratio of the as-synthesized β-TCP sample, as well as with the elemental compositions determined by ICP-MS, which underlines the quantitative accuracy of the refinement model. Deprotonization and the precipitation of pyrophosphate was also observed when applying the refinement model to calcined synthetic Mg-whitlockite, which is in agreement with previous studies (Adcock *et al.*, 2014 ▸).

The stoichiometry of the β-TCP platelets was independent of numerous synthesis parameters. Notably, neither the temperature nor the precursor Ca/P ratio nor the total concentration had an effect on the final Ca/P ratio in the crystals, indicating that the Ca deficiency is not the result of limited Ca ion supply or diffusion during crystallization. This conclusion is in line with the fact that the Ca/P ratio was identical in platelets produced in glycerol, a solvent exhibiting a 30-fold lower ionic mobility compared with ethylene glycol due to its higher viscosity (Kameche *et al.*, 2005 ▸). Also, since the final stoichiometry was independent of the reaction time (varying from 1 min to 24 h), the Ca deficiency cannot be a result of Ca diffusing out of the crystals after their formation. Overall, these findings strongly suggest that the platelet structure is a thermodynamically stable and non-kinetically limited phase. This is in contrast with the precipitation of CDHA in aqueous solutions where the Ca/P ratio can vary between 1.5 and 1.67 and is known to increase with increasing precursor Ca/P ratio, reaction temperature and time (Raynaud *et al.*, 2002 ▸; Ishikawa *et al.*, 1993 ▸; Vallet-Regí *et al.*, 1997 ▸). In particular, the Ca/P ratio in CDHA gradually increases towards a thermodynamically more stable value (closer to 1.67) when incubated for longer time periods (Vallet-Regí *et al.*, 1997 ▸). Moreover, the Ca/P ratio in CDHA varies with the solution pH (Vallet-Regí *et al.*, 1997 ▸), whereas the acidity of ethylene glycol solutions did not influence the Ca/P ratio in the platelets, even when approaching the stability region of the monetite phase. Interestingly, several of these synthesis parameters had a major effect on the platelet geometry, with the diameter and aspect ratio varying from 0.2 to 2 µm and from 1 to 14, respectively, as reported previously (Galea *et al.*, 2013 ▸, 2014 ▸). Given this large range of surface-to-volume ratios in samples with almost identical Ca/P ratios, the Ca deficiency is likely homogeneously distributed throughout the crystals. This finding is consistent with a previous study reporting that the center and edge region of the single crystals were crystallographically identical (Tao *et al.*, 2008 ▸).

Owing to their controllable geometry and non-agglomerating properties, the platelets described in this study are of interest as fillers in nano-structured load-bearing composites or, at lower aspect ratios, may enhance the flowability of CaP pastes (Galea *et al.*, 2013 ▸). The success of a bone substitute material relies on the solubility and degradation kinetics in aqueous environments, which are dependent on the crystal structure. In (CD)HA, a lower Ca/P ratio, *i.e.* a higher degree of hydrogen substitution, is known to lead to a higher dissolution rate and an earlier precipitation of an apatite surface layer (Radin & Ducheyne, 1993 ▸; Mavropoulos *et al.*, 2003 ▸). In β-TCP and whitlockite, Mg plays an important role in the thermodynamic stability. Specifically, substitution of 8 mol% Ca for Mg in β-TCP increases the thermal stability from 1423 K up to 1873 K (Enderle *et al.*, 2005 ▸), while at low temperature Mg reduces the solubility of whitlockite below that of β-TCP and CDHA (Hamad & Heughebaert, 1986 ▸). The stabilizing effect of Mg dominates any additional change in solubility resulting from the hydrogen substitution. On the other hand, the structure presented in this study is free of Mg and thus allows for an isolated analysis of the effect of hydrogen substitution on the solubility of β-TCP, which will be a priority in future studies.

Since β-TCP bone grafts often require more than a year for complete resorption (Van Der Pol *et al.*, 2010 ▸), a material with a higher degradation rate than stoichiometric β-TCP may provide a clinical benefit in orthopaedic applications. Monetite and brushite materials exhibit very fast resorption but are associated with acidic pH changes when transforming into HA due to the presence of HPO_4_^2−^ groups, which limits the volume of their application. Since HPO_4_^2−^ groups represent only a fraction of the phosphate groups in platelets, weaker pH changes are expected compared with monetite or brushite. Therefore, a more soluble hydrogen-substituted β-TCP material may provide a faster resorbing, highly biocompatible alternative to stoichiometric β-TCP bone graft substitutes.

## 5. Conclusions   

This study elucidated the crystal structure of β-TCP platelets precipitated in ethylene glycol. Rietveld refinement of XRD patterns indicated sub-occupied Ca4 and O2 atomic sites compared with the published β-TCP crystal model. In contrast, a model adopted from the whitlockite structure, where Ca is partly substituted by H along with the inversion of P1O_4_ tetrahedra, resulted in precise and reproducible refinements with stable convergence towards a Ca/P ratio of 1.443 ± 0.003 (*n* = 36) and was corroborated by the presence of HPO_4_^2−^ absorptions in FTIR spectra. Calcination of the platelets led to phase separation into H-free β-TCP (Ca/P = 1.5) and β-CPP (Ca/P = 1.0) which served as a quantitative verification of the initially refined β-TCP Ca/P ratio. Specifically, the global Ca/P ratio closely matched the β-TCP Ca/P ratio before calcination (ΔCa/P = 0.003) and the chemical composition measured by ICP-MS (ΔCa/P = 0.003). The Ca/P ratio was independent of synthesis parameters including temperature, time, pH, precursor Ca/P ratio and concentration, which indicates a thermodynamically stable phase. These findings describe for the first time a hydrogen-substituted β-TCP structure or, in other words, an Mg-free whitlockite and thus raise the question of the role of hydrogen substitution in β-TCP solubility.

## 6. Related literature   

References cited in the supporting information include: Cerruti *et al.* (2014 ▸), Nuevo *et al.* (2006 ▸), Ping *et al.* (2001 ▸) and Tortet (1997 ▸).

## Supplementary Material

Crystal structure: contains datablock(s) global, 150212-01-betaTCP, 150212-01-Dicalciumphosphate, 150212-01-Chlorapatite, 150212-02-betaTCP, 150212-02-Dicalciumphosphate, 150212-02-Chlorapatite, 150213-01-betaTCP, 150213-01-Dicalciumphosphate, 150213-01-Chlorapatite, 150213-02-betaTCP, 150213-02-Dicalciumphosphate, 150213-02-Chlorapatite, 150216-01-betaTCP, 150216-01-Dicalciumphosphate, 150216-01-Chlorapatite, 150216-02-betaTCP, 150216-02-Dicalciumphosphate, 150216-02-Chlorapatite, 150217-01-betaTCP, 150217-01-Dicalciumphosphate, 150217-01-Chlorapatite, 150220-01-betaTCP, 150220-01-Dicalciumphosphate, 150220-01-Chlorapatite, 150220-02-betaTCP, 150220-02-Dicalciumphosphate, 150220-02-Chlorapatite, 150305-04-betaTCP, 150305-04-Chlorapatite, 150306-01-betaTCP, 150306-01-Dicalciumphosphate, 150306-01-Chlorapatite, 150306-02-betaTCP, 150306-02-Dicalciumphosphate, 150306-02-Chlorapatite, 150309-02-betaTCP, 150309-02-Dicalciumphosphate, 150309-02-Chlorapatite, 150310-01-betaTCP, 150310-01-Dicalciumphosphate, 150310-01-Chlorapatite, 150311-02-betaTCP, 150311-02-Dicalciumphosphate, 150311-02-Chlorapatite, 150316-01-betaTCP, 150316-01-Dicalciumphosphate, 150316-01-Chlorapatite, 150317-01-betaTCP, 150317-01-Dicalciumphosphate, 150317-01-Chlorapatite, 150317-02-betaTCP, 150317-02-Dicalciumphosphate, 150317-02-Chlorapatite, 150318-01-betaTCP, 150318-01-Dicalciumphosphate, 150318-01-Chlorapatite, 150323-01-betaTCP, 150323-01-Dicalciumphosphate, 150323-01-Chlorapatite, 150324-01-betaTCP, 150324-01-Dicalciumphosphate, 150324-01-Chlorapatite, 150325-02-betaTCP, 150325-02-Dicalciumphosphate, 150325-02-Chlorapatite, 150508-01-betaTCP, 150508-01-Dicalciumphosphate, 150508-01-Chlorapatite, 150511-02-betaTCP, 150511-02-Dicalciumphosphate, 150511-02-Chlorapatite, 150512-01-betaTCP, 150512-01-Dicalciumphosphate, 150512-01-Chlorapatite, 150512-02-betaTCP, 150512-02-Dicalciumphosphate, 150512-02-Chlorapatite, 150528-01-betaTCP, 150528-01-Dicalciumphosphate, 150528-01-Chlorapatite, 150529-02-betaTCP, 150529-02-Dicalciumphosphate, 150529-02-Chlorapatite, 150601-01-betaTCP, 150601-01-Dicalciumphosphate, 150601-01-Chlorapatite, 150601-02-betaTCP, 150601-02-Dicalciumphosphate, 150601-02-Chlorapatite, 150602-01-betaTCP, 150602-01-Dicalciumphosphate, 150602-01-Chlorapatite, 150608-01-betaTCP, 150608-01-Dicalciumphosphate, 150608-01-Chlorapatite, 150608-02-betaTCP, 150608-02-Dicalciumphosphate, 150608-02-Chlorapatite, 150609-02-betaTCP, 150609-02-Dicalciumphosphate, 150609-02-Chlorapatite, 150610-01-betaTCP, 150610-01-Dicalciumphosphate, 150610-01-Chlorapatite, 150610-02-betaTCP, 150610-02-Dicalciumphosphate, 150610-02-Chlorapatite, 150623-01-betaTCP, 150623-01-betaCPP, 150624-01-betaTCP, 150624-01-betaCPP, 150624-02-betaTCP, 150624-02-betaCPP, 150812-02-betaTCP. DOI: 10.1107/S2052520616015675/bm5088sup1.cif

Rietveld powder data: contains datablock(s) 150212-01. DOI: 10.1107/S2052520616015675/bm5088150212-01-betaTCPsup2.rtv

Rietveld powder data: contains datablock(s) 150212-02. DOI: 10.1107/S2052520616015675/bm5088150212-02-betaTCPsup3.rtv

Rietveld powder data: contains datablock(s) 150213-01. DOI: 10.1107/S2052520616015675/bm5088150213-01-betaTCPsup4.rtv

Rietveld powder data: contains datablock(s) 150213-02. DOI: 10.1107/S2052520616015675/bm5088150213-02-betaTCPsup5.rtv

Rietveld powder data: contains datablock(s) 150216-01. DOI: 10.1107/S2052520616015675/bm5088150216-01-betaTCPsup6.rtv

Rietveld powder data: contains datablock(s) 150216-02. DOI: 10.1107/S2052520616015675/bm5088150216-02-betaTCPsup7.rtv

Rietveld powder data: contains datablock(s) 150217-01. DOI: 10.1107/S2052520616015675/bm5088150217-01-betaTCPsup8.rtv

Rietveld powder data: contains datablock(s) 150220-01. DOI: 10.1107/S2052520616015675/bm5088150220-01-betaTCPsup9.rtv

Rietveld powder data: contains datablock(s) 150220-02. DOI: 10.1107/S2052520616015675/bm5088150220-02-betaTCPsup10.rtv

Rietveld powder data: contains datablock(s) 150305-04. DOI: 10.1107/S2052520616015675/bm5088150305-04-betaTCPsup11.rtv

Rietveld powder data: contains datablock(s) 150306-01. DOI: 10.1107/S2052520616015675/bm5088150306-01-betaTCPsup12.rtv

Rietveld powder data: contains datablock(s) 150306-02. DOI: 10.1107/S2052520616015675/bm5088150306-02-betaTCPsup13.rtv

Rietveld powder data: contains datablock(s) 150309-02. DOI: 10.1107/S2052520616015675/bm5088150309-02-betaTCPsup14.rtv

Rietveld powder data: contains datablock(s) 150310-01. DOI: 10.1107/S2052520616015675/bm5088150310-01-betaTCPsup15.rtv

Rietveld powder data: contains datablock(s) 150311-02. DOI: 10.1107/S2052520616015675/bm5088150311-02-betaTCPsup16.rtv

Rietveld powder data: contains datablock(s) 150316-01. DOI: 10.1107/S2052520616015675/bm5088150316-01-betaTCPsup17.rtv

Rietveld powder data: contains datablock(s) 150317-01. DOI: 10.1107/S2052520616015675/bm5088150317-01-betaTCPsup18.rtv

Rietveld powder data: contains datablock(s) 150317-02. DOI: 10.1107/S2052520616015675/bm5088150317-02-betaTCPsup19.rtv

Rietveld powder data: contains datablock(s) 150318-01. DOI: 10.1107/S2052520616015675/bm5088150318-01-betaTCPsup20.rtv

Rietveld powder data: contains datablock(s) 150323-01. DOI: 10.1107/S2052520616015675/bm5088150323-01-betaTCPsup21.rtv

Rietveld powder data: contains datablock(s) 150324-01. DOI: 10.1107/S2052520616015675/bm5088150324-01-betaTCPsup22.rtv

Rietveld powder data: contains datablock(s) 150325-02. DOI: 10.1107/S2052520616015675/bm5088150325-02-betaTCPsup23.rtv

Rietveld powder data: contains datablock(s) 150508-01. DOI: 10.1107/S2052520616015675/bm5088150508-01-betaTCPsup24.rtv

Rietveld powder data: contains datablock(s) 150511-02. DOI: 10.1107/S2052520616015675/bm5088150511-02-betaTCPsup25.rtv

Rietveld powder data: contains datablock(s) 150512-01. DOI: 10.1107/S2052520616015675/bm5088150512-01-betaTCPsup26.rtv

Rietveld powder data: contains datablock(s) 150512-02. DOI: 10.1107/S2052520616015675/bm5088150512-02-betaTCPsup27.rtv

Rietveld powder data: contains datablock(s) 150528-01. DOI: 10.1107/S2052520616015675/bm5088150528-01-betaTCPsup28.rtv

Rietveld powder data: contains datablock(s) 150529-02. DOI: 10.1107/S2052520616015675/bm5088150529-02-betaTCPsup29.rtv

Rietveld powder data: contains datablock(s) 150601-01. DOI: 10.1107/S2052520616015675/bm5088150601-01-betaTCPsup30.rtv

Rietveld powder data: contains datablock(s) 150601-02. DOI: 10.1107/S2052520616015675/bm5088150601-02-betaTCPsup31.rtv

Rietveld powder data: contains datablock(s) 150602-01. DOI: 10.1107/S2052520616015675/bm5088150602-01-betaTCPsup32.rtv

Rietveld powder data: contains datablock(s) 150608-01. DOI: 10.1107/S2052520616015675/bm5088150608-01-betaTCPsup33.rtv

Rietveld powder data: contains datablock(s) 150608-02. DOI: 10.1107/S2052520616015675/bm5088150608-02-betaTCPsup34.rtv

Rietveld powder data: contains datablock(s) 150609-02. DOI: 10.1107/S2052520616015675/bm5088150609-02-betaTCPsup35.rtv

Rietveld powder data: contains datablock(s) 150610-01. DOI: 10.1107/S2052520616015675/bm5088150610-01-betaTCPsup36.rtv

Rietveld powder data: contains datablock(s) 150610-02. DOI: 10.1107/S2052520616015675/bm5088150610-02-betaTCPsup37.rtv

Rietveld powder data: contains datablock(s) 150623-01. DOI: 10.1107/S2052520616015675/bm5088150623-01-betaTCPsup38.rtv

Rietveld powder data: contains datablock(s) 150624-01. DOI: 10.1107/S2052520616015675/bm5088150624-01-betaTCPsup39.rtv

Rietveld powder data: contains datablock(s) 150624-02. DOI: 10.1107/S2052520616015675/bm5088150624-02-betaTCPsup40.rtv

Rietveld powder data: contains datablock(s) 150812-02. DOI: 10.1107/S2052520616015675/bm5088150812-02-betaTCPsup41.rtv

Supplementary methods and results. DOI: 10.1107/S2052520616015675/bm5088sup42.pdf

CCDC references: 1508203, 1508204, 1508205, 1508206, 1508207, 1508208, 1508209, 1508210, 1508211, 1508212, 1508213, 1508214, 1508215, 1508216, 1508217, 1508218, 1508219, 1508220, 1508221, 1508222, 1508223, 1508224, 1508225, 1508226, 1508227, 1508228, 1508229, 1508230, 1508231, 1508232, 1508233, 1508234, 1508235, 1508236, 1508237, 1508238, 1508239, 1508240, 1508241, 1508242, 1508243, 1508244, 1508245, 1508246, 1508247, 1508248, 1508249, 1508250, 1508251, 1508252, 1508253, 1508254, 1508255, 1508256, 1508257, 1508258, 1508259, 1508260, 1508261, 1508262, 1508263, 1508264, 1508265, 1508266, 1508267, 1508268, 1508269, 1508270, 1508271, 1508272, 1508273, 1508274, 1508275, 1508276, 1508277, 1508278, 1508279, 1508280, 1508281, 1508282, 1508283, 1508284, 1508285, 1508286, 1508287, 1508288, 1508289, 1508290, 1508291, 1508292, 1508293, 1508294, 1508295, 1508296, 1508297, 1508298, 1508299, 1508300, 1508301, 1508302, 1508303, 1508304, 1508305, 1508306, 1508307, 1508308, 1508309, 1508310, 1508311, 1508312, 1508313, 1508314, 1508315, 1508316

## Figures and Tables

**Table 1:** XRD data acquisition parameters, refinement statistics, space group and unit-cell constants

Radiation, wavelength (Å)	Cu *K*α, 1.540598
2θ range (°)	5–60
Step scan increment (°2θ)	0.012
	
Refinement statistics†	
*R*_wp_ (%), defined previously (McCusker *et al.*, 1999 ▸)	6.4 ± 0.6
*R*_exp_ (%) (McCusker *et al.*, 1999 ▸)	5.9 ± 0.6
χ^2^ = (*R*_wp_/*R*_exp_)^2^, defined previously (Toby, 2006 ▸)	1.17 ± 0.06
	
Unit cell†	
Space group	*R*3*c*
*a* (Å)	10.471 ± 0.006
*c* (Å)	37.371 ± 0.012

† Mean values and standard deviations from 36 samples.

**Table 2:** Site occupancies and atomic coordinates for the original and mirrored (H)P1O_4_ tetrahedron

		Atomic coordinates† (as fractions of the unit-cell constants)		
Atom	Occupancy†	*x*	*y*	*z*
Ca4	0.100 ± 0.021	0	0	−0.0843 ± 0.0024
				
Original P1O_4_ tetrahedron				
P1	0.199 ± 0.041	0	0	0
O1	1	0.0126 ± 0.0037	−0.1345 ± 0.0041	−0.0104 ± 0.0009
O2	0.199 ± 0.041	0	0	0.0401 ± 0.0000
				
Mirrored HP1′O_4_ tetrahedron				
P1′	0.801 ± 0.041	0	0	−0.0209 ± 0.0018
O1	1	0.0126 ± 0.0037	−0.1345 ± 0.0041	−0.0104 ± 0.0009
O2′	0.801 ±0.041	0	0	−0.0610 ± 0.0018
H	0.801 ± 0.041	0	0	−0.0862 ± 0.0018

† Mean values and standard deviations from 36 samples.

**Table 3:** Comparison of the β-TCP Ca/P molar ratio, phase weight fractions (*f*_w_) and overall (over all phases, excluding chlorapatite) Ca/P ratio before and after calcination Mean values and standard deviations (SD) from three samples (synthesized using standard conditions or 443 K), determined by XRD and Rietveld refinement with the hydrogen-substituted model.

		Ca/P	*f*_w_ (%)			Ca/P
		β-TCP	β-TCP	Monetite	β-CPP	Overall
Pre-calcination	Mean (*n* = 3)	1.445	98.4	1.6	–	1.437
	SD	± 0.001	± 0.8	± 0.8	–	± 0.003
Post calcination	Mean (*n* = 3)	1.500	89.9	–	10.1	1.440
	SD	± 0.000	± 1.1	–	± 1.1	± 0.006
			Difference (post − pre calc.)			0.003
					SD	± 0.003

**Table 4:** The chemical composition measured by ICP before and after calcination and comparison with the overall Ca/P ratio determined by XRD (see Table 3 ▸) Mean molar ratios and standard deviations (SD) from three samples synthesized using standard conditions or 443 K.

		ICP				Δ(ICP − XRD)
		(Ca/P)_ICP,corr_ †	(Na/P)_ICP_	(Mg/P)_ICP_	(Ca_eq_/P)_ICP,corr_ ‡	(Ca_eq_/P)_ICP,corr_ − (Ca/P)_XRD_
Pre calc.	Mean (*n* = 3)	1.380	0.018	0.001	1.391	−0.047
	SD	± 0.010	± 0.004	± 0.000	± 0.009	± 0.006
Post calc.	Mean (*n* = 3)	1.432	0.007	0.002	1.437	−0.003
	SD	± 0.001	± 0.000	± 0.001	± 0.001	± 0.007

† Corrected as described in §2.4.

‡ Ca-equivalent ratio, to account for substitutional cations as defined in §3.4.
