# Supplementary material for: Hydrogen-substituted β-tricalcium phosphate synthesized in organic media
Source: Acta Crystallogr B Struct Sci Cryst Eng Mater. 2016 Dec 1;72(Pt 6):875–84. doi: 10.1107/S2052520616015675 (PMC5134762; doi:10.1107/S2052520616015675)
Supplement: Supplementary file 42 [file b-72-00875-sup42.pdf]

STRUCTURAL SCIENCE
CRYSTAL ENGINEERING
MATERIALS

Volume 72 (2016)

Supporting information for article:

Hydrogen-substituted β-tricalcium phosphate synthesized in organic media

Christoph Stähli, Jürg Thüring, Laëtitia Galea, Solène Tadier, Marc Bohner and Nicola Döbelin

S1. Methods

S1.1. Details on the β-TCP platelet synthesis

An ethylene glycol ($C_2H_6O_2$, Reag.Ph.Eur., VWR, Switzerland) solution containing phosphate ions ($Na_2HPO_4 \cdot 2H_2O$, purum p.a., Fluka, Switzerland; or H_3PO_4, 85%, puriss. pa., ACS, Fluka, Switzerland) was mixed with an ethylene glycol solution containing calcium ions ($CaCl_2 \cdot 2H_2O$, Reag.Ph.Eur., MERCK, Germany). The acidity was adjusted by the addition of NaOH (NaOH, puriss. p.a., Fluka, Switzerland) to the phosphate solution. In the batch reactor, the phosphate solution was poured into the pre-heated calcium solution and kept at 90 to 170°C under intensive stirring for 70 s to 24 h. In the tubular reactor, the phosphate and calcium solutions were first pumped through a heating bath at 90°C in two separate tubes (inner diameter: 1/16 in, fluorinated ethylene propylene; PKM SA, Switzerland) and subsequently combined in a static mixer (Nordson, USA) and pumped through a second heating bath at 150°C for approximately 20 min to allow for precipitation. The resulting platelet suspension was centrifuged at 4000 rpm for 30 min and the precipitate was washed by successive dispersion and centrifugation in ethanol (C_2H_6O, absolute 99.8%, Grogg, Switzerland), demineralized water and again in ethanol, before drying at 30°C under vacuum for 24 h.
The synthesis conditions are detailed in **Error! Reference source not found.** namely standard conditions (applied for all analyses unless otherwise indicated) and conditions with individually varied parameters to study the effect of the latter on the crystal structure. Note that, since the pH could not be accurately measured in ethylene glycol, a measure of the acidity was defined as the difference between the H^+ and OH^- ionic concentrations introduced into the solution, in the form of NaOH, Na_2HPO_4 and/or H_3PO_4, normalized by the total precursor concentration, i.e. ($[H^+]$-$[OH^-]$)/([Ca]+[P]). To produce Mg-containing platelets, 0.5 or 1 mol% of the Ca ions were replaced by Mg ions ($MgCl_2 \cdot 6H_2O$, Ph.Eur., MERCK, Germany). Glycerol was used as an alternative solvent (glycerol, anhydr. for synth., MERCK, Germany). **Error! Reference source not found.** links the numbered synthesis conditions to the corresponding dataset name used in the CIF files.

Table S1 Synthesis parameters corresponding to the numbered conditions in Fig. 4, i.e. standard condition (STD) and conditions with varied parameters in each sub-group (marked in bold).

Condition no.	STD	A1, A2, A3, A4	B1, B2	C1, C2	D1, D2, D3	E1, E2	F1, F2	G
Reaction temperature [°C]	**150**	**90, 110, 130, 170**	150	150	150	150	150	150
Precursor Ca/P ratio (molar)	**1.5**	1.5	**1.1, 2**	1.5	1.5	1.5	1.5	1.67
Precursor conc. (Ca+P) [mM]	**16**	16	16	**32, 48**	32	16	16	16
Acidity: ([H]-[OH])/([Ca]+[P])	**0.27**	0.27	0.34, 0.20	0.36	**0.42, 0.30, 0.19**	0.27	0.27	0.23
Mg doping (Mg/Ca [mol%])	**0**	0	0	0	0	**0.5, 1.7**	0	0
Reaction time	**24 h**	24 h	1.5 h	24, 0.3 h	0.3 h	24 h	**70, 90 s**	1.5 h
Solvent type	**Et.Gly.**	Et.Gly.	Et.Gly.	Et.Gly.	Et.Gly.	Et.Gly.	Et.Gly.	**Glyc.**
Reactor type	Batch	Batch	Batch	Batch, Tub.	Tub.	Batch	Batch	Batch
No of samples per condition	4	2, 2, 2, 2	3, 3	3, 1	3, 3, 3	1, 1	1, 1	2

Table S2 List of samples analyzed by Rietveld refinement of XRD patterns, matching the numbered synthesis conditions (see **Error! Reference source not found.**) with the corresponding dataset name in the CIF files.

Sample code (synthesis condition _ synthesis repeat)	Name of dataset in CIF files
As-made β-TCP platelets	
STD_1	150512-02
STD_2	150317-01
STD_3	150601-02
STD_4	150529-02
A1_1	150318-01
A2_1	150311-02
A2_2	150325-02
A3_1	150316-01
A3_2	150508-01
A4_1	150317-02
A4_2	150528-01
B1_1	150305-04
B1_2	150306-02
B1_3	150601-01
B2_1	150306-01
B2_2	150309-02
B2_3	150602-01
C1_1	150609-02
C1_2	150610-01
C1_3	150610-02
C2_1	150310-01
D1_1	150213-01
D1_2	150216-02
D1_3	150220-02
D2_1	150212-02
D2_2	150216-01
D2_3	150220-01
D3_1	150212-01
D3_2	150213-02
D3_3	150217-01
E1_1	150512-01
E2_1	150511-02
F1_1	150608-02
F2_1	150608-01
G_1	150323-01
G_2	150324-01
Calcined β-TCP platelets	
STD_1(calc.)	150623-01
A4_1(calc.)	150624-01
A4_2(calc.)	150624-02
Synthetic whitlockite	
WHITL	150812-02

S1.2. Scanning electron microscopy

The scanning electron microscopy image in **Error! Reference source not found.** was obtained on a Zeiss EVO MA25 instrument using a working distance of 8 mm and an acceleration tension of 20 kV, following sputter-coating with Pt for 30 s.

S1.3. Attenuated total reflection (ATR)-FTIR

In addition to the transmission FTIR analysis (detailed in section 2.3), the samples were analyzed in the attenuated total reflection (ATR) mode, by means of a diamond crystal on the Bruker Lumos IR spectrometer. Spectra were acquired between 400 and 4000 cm^{-1} at a resolution of 4 cm^{-1} and 64 accumulations.

S2. Results

S2.1. Morphology

The hexagonal morphology of a representative β-TCP platelet sample (synthesized under standard conditions; see **Error! Reference source not found.**) is shown in **Error! Reference source not found.**. The detailed relationships between synthesis conditions and geometric features have been described previously (Galea *et al.*, 2013; Galea *et al.*, 2014).

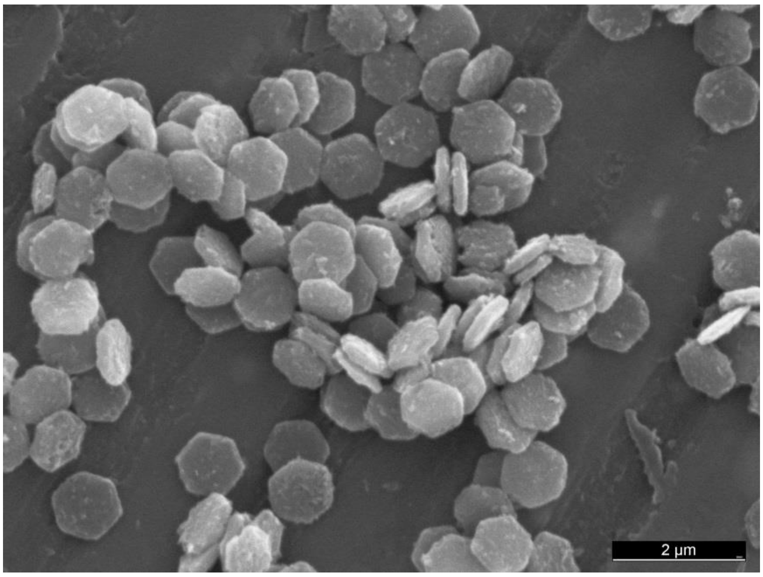

Figure S1 Scanning electron microscopy image showing the morphology of β-TCP platelets synthesized at standard conditions.

S2.2. FTIR analysis

The full wavenumber range of transmission FTIR spectra of sintered β-TCP and β-TCP platelets is provided **Error! Reference source not found.**. The broad band at 3700 to 3200 cm^{-1} and the absorption at 1630 cm^{-1} are attributable to OH^- groups (Ping *et al.*, 2001) and H_2O molecules (Cerruti *et al.*, 2003) respectively, resulting from varying degrees of residual moisture in the KBr pellets used for transmission FTIR analysis. Note that this moisture cannot account for the HPO_4^{2-} absorption observed at 875 cm^{-1}, since the latter was also observed in ATR-FTIR spectra which were free of water-related absorptions (see **Error! Reference source not found.**). The peak at 2350 cm^{-1} is due to slight variations in the gaseous CO_2 concentration between the different analyses (Nuevo *et al.*, 2006). Finally, the small bands close to 2900 cm^{-1} were attributed to organic contaminations (C-H bonds) in the KBr pellets. Note that this signal was not detected in ATR-FTIR spectra (**Error! Reference source not found.**b).

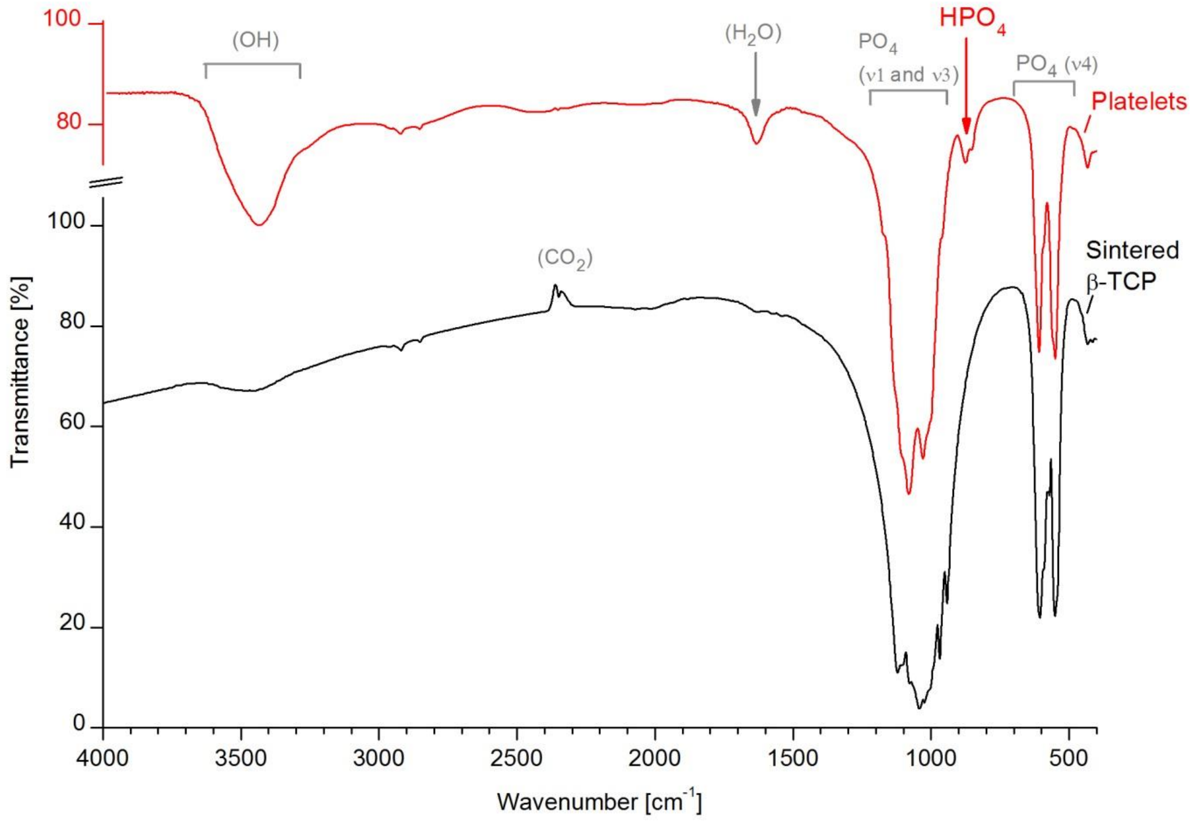

Figure S2 Full wavenumber range of transmission FTIR spectra of sintered β-TCP and β-TCP platelets. (The wavenumber region showing phosphate absorption is detailed in Fig. 2). The absorptions at 3700-3200 cm^{-1} and 1630 cm^{-1} are attributable to residual moisture in the KBr pellets while the peak at 2350 cm^{-1} results from gaseous CO_2.

ATR-FTIR spectra of commercially available monetite as well as β-TCP platelets containing variable fractions of monetite platelets are provided in **Error! Reference source not found.**. These spectra were compared amongst each other in order to ensure that the peak at 875 cm^{-1}, assigned to HPO_4^{2-} groups in the β-TCP structure, does not result from the monetite ($CaHPO_4$) platelets, a by-product of the synthesis obtained especially at lower temperatures. The spectrum of pure monetite reveals a broad

band with a maximum at 890 cm^{-1}, attributable to the P-O(H) stretching mode in HPO_4^{2-} groups (Tortet *et al.*, 1997), which overlaps indeed with the band at 875 cm^{-1} in β-TCP platelets. However, monetite exhibits several other absorptions which are not present in β-TCP, namely between 1300 and 1450 cm^{-1} (P-O-H in-plane bending) (Tortet *et al.*, 1997) and close to 400 cm^{-1} (O-P-O bending; partly cut off by the lower spectral range limit) (Tortet *et al.*, 1997). Thus, if the HPO_4^{2-} band of the purest β-TCP sample (1 wt% monetite) was due to the monetite platelets, a strong absorption at 400 cm^{-1} would have to be present, which is not the case. Moreover, the intensity of the absorption at 875 cm^{-1} does not converge to zero as the monetite fraction approaches zero. Therefore, the band at 875 cm^{-1} in β-TCP platelets – as well as the side peak at 855 cm^{-1} which is not observed in monetite – must result predominantly from HPO_4^{2-} groups in the β-TCP structure and is thus corroborate the Rietveld refinement findings.

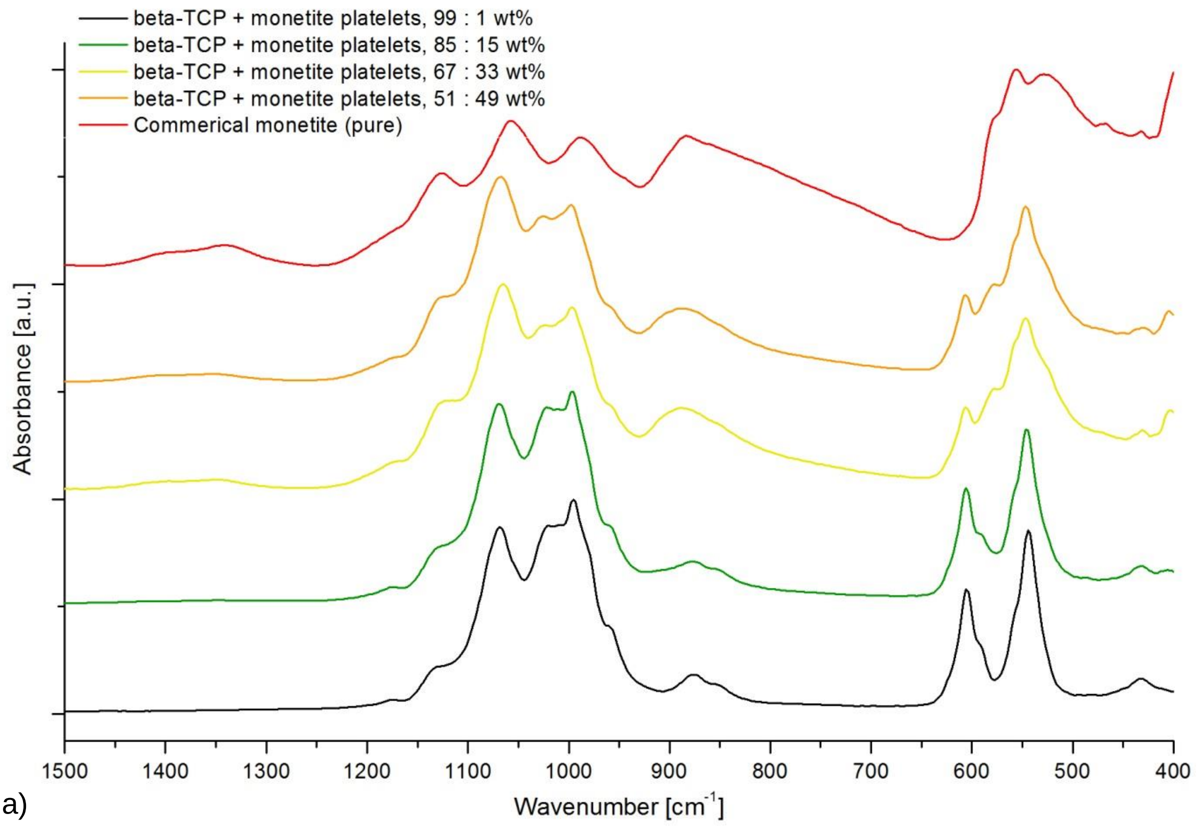

a)

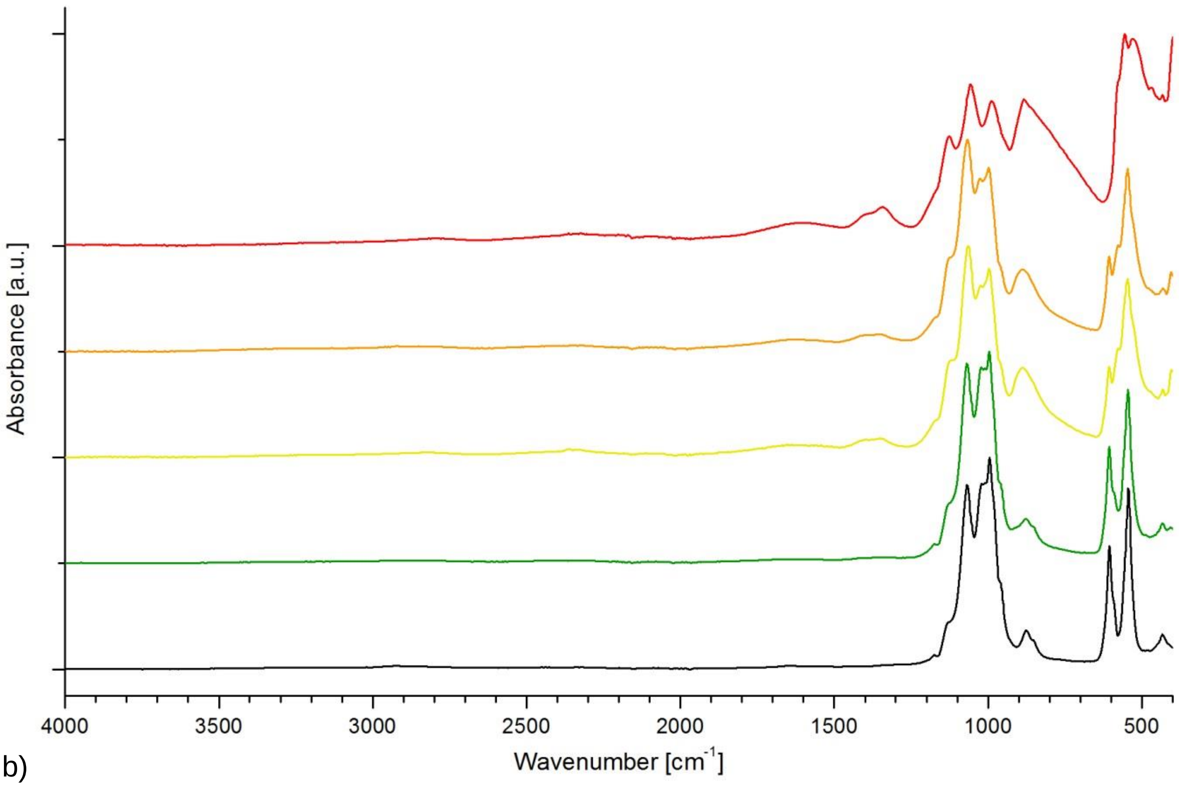

b)

Figure S3 ATR-FTIR spectra of commercial monetite and β-TCP platelets containing increasing amounts of monetite platelets. (a) Wavenumber region of phosphate absorptions and (b) full wavenumber range.

S2.3. Effect of synthesis conditions - statistical analysis

One-way ANOVA followed by Bonferroni means comparison indicated no significant difference ($p>0.05$) in the β-TCP Ca/P (or (Ca+Mg)/P ratio) between any of the synthesis conditions for which at least two samples were analyzed. Moreover, the differences between the standard condition and conditions with only one analyzed sample were within two times the estimated standard deviation (ESD) calculated by the refinement algorithm (error bars in Fig. 4). Note that the large ESDs obtained in conditions A1 and C2 can be explained by the high monetite fractions in the sample (data not shown).

For some conditions, more than one synthesis parameter was varied simultaneously. Specifically, samples in conditions C2 and D1 to D3 were produced in the tubular reactor instead of the batch reactor (see **Error! Reference source not found.**). However, a significant effect of the reactor type on the Ca/P ratio can be ruled out by comparing condition C1 to group D. Similarly, while the acidity and reaction time are inconsistent in some conditions, groups D and F demonstrate that these parameters have no significant effect.

References

Cerruti, M., Magnacca, G., Bolis, V. & Morterra, C. (2003). *J. Mater. Chem.* **13**, 1279-1286.
Galea, L., Bohner, M., Thuering, J., Doebelin, N., Aneziris, C. G. & Graule, T. (2013). *Biomaterials* **34**, 6388-6401.
Galea, L., Bohner, M., Thuering, J., Doebelin, N., Ring, T. A., Aneziris, C. G. & Graule, T. (2014). *Acta Biomater.* **10**, 3922-3930.
Nuevo, M., Meierhenrich, U. J., Muñoz Caro, G. M., Dartois, E., D'Hendecourt, L., Deboffle, D., Auger, G., Blanot, D., Bredehöft, J. H. & Nahon, L. (2006). *Astron. Astrophys.* **457**, 741-751.
Ping, Z. H., Nguyen, Q. T., Chen, S. M., Zhou, J. Q. & Ding, Y. D. (2001). *Polymer* **42**, 8461-8467.
Tortet, L., Gavarri, J. R., Nihoul, G. & Dianoux, A. J. (1997). *J. Solid State Chem.* **132**, 6-16.
